# Glutathione-dependent enzymes in the follicular fluid of the first-retrieved oocyte and their impact on oocyte and embryos in polycystic ovary syndrome: A cross-sectional study

**DOI:** 10.18502/ijrm.v13i6.7283

**Published:** 2020-06-30

**Authors:** Fatemeh Zal, Pardis Ahmadi, Maryam Davari, Fatemeh Khademi, Mojgan Akbarzadeh Jahromi, Zahra Anvar, Bahia Namavar Jahromi

**Affiliations:** ^1^Department of Biochemistry, School of Medicine, Shiraz University of Medical Sciences, Shiraz, Iran.; ^2^Infertility Research Center, Shiraz University of Medical Sciences, Shiraz, Iran.; ^3^Department of Obstetrics and Gynecology, School of Medicine, Shiraz University of Medical Sciences, Shiraz, Iran.; ^4^IVF Section, Ghadir-Mother and Child Hospital of Shiraz, Shiraz, Iran.; ^5^Department of Pathology, Maternal-Fetal Medicine Research Center, Shiraz University of Medical Sciences, Shiraz, Iran.

**Keywords:** In vitro fertilization, Glutathione, Antioxidant, Oocyte, Embryo.

## Abstract

**Background:**

Oxidative stress and GSH-dependent antioxidant system plays a key role in the pathogenesis of polycystic ovary syndrome (PCOS).

**Objective:**

We compared glutathione peroxidase (GPx) and glutathione reductase activities and reduced glutathione (GSH) levels in serum and follicular fluid (FF) of the first-retrieved follicle and their impact on quality of oocyte and embryo in PCOS women undergoing IVF.

**Materials and Methods:**

This cross sectional study was conducted on 80 pairs of blood samples and FF of the first-retrieved follicle from PCOS women, at the Infertility center of Ghadir Mother and Child Hospital. The mean activity of GPx and GR, also GSH levels in the serum and FF were compared to the quality of the first follicle and resultant embryo.

**Results:**

Retrieved oocytes included 53 (66.25%) MII, 17 (21.25%) MI, and 10 (12.5%) germinal vesicles; after IVF 42 (52.50%) embryos with grade I and 11 (13.75%) with grade II were produced. The mean values for all three antioxidants were higher in the FF compared to serum (p < 0.001). Also all of the mean measured levels were significantly higher in the FF of the MII oocytes compared to that of oocytes with lower grades (p = 0.012, 0.006 and 0.012, respectively). The mean GPX activity and GSH levels were significantly higher in the serum (p = 0.016 and 0.012, respectively) and FF (p = 0.001 for both) of the high-quality grade I embryos.

**Conclusion:**

GSH-dependent antioxidant system functions more efficiently in the FF of oocytes and embryos with higher quality.

## 1. Introduction

Polycystic ovary syndrome (PCOS) as the most common endocrine disorder affects 6-8% of reproductive-age women. “According to the revised Rotterdam 2003 criteria is diagnosed when two of the following three criteria are present: (1) oligo- or anovulation, (2) clinical and/or biochemical signs of hyperandrogenism, and (3) polycystic ovaries on ultrasound (1, 2)". In addition to the hormonal and metabolic dysfunctions in PCOS, excessive production of reactive oxygen species (ROS) is also considered to be implicated in pathophysiology of the syndrome (3).

Oxidative stress (OS) refers to an imbalance between produced ROS and detoxifying capacity of antioxidants (4). It is suggested that cellular ROS play an important role in several diseases including atherosclerosis, diabetes, metabolic syndrome, obesity, and impaired reproductive disorders like PCOS (5). How PCOS is related to OS could be explained by the role of antioxidants in folliculogenesis, ovulation, and some other ovarian features. Any disequilibrium in these mechanisms may impair the female reproductive system. Moreover, OS may affect the results of assisted reproductive technologies (ART) (6).

Glutathione (GSH) is an important intracellular antioxidant. It can be converted to its oxidized form (GSSG) by glutathione peroxidase (GPx) when functioning as a scavenger; it is converted back to its reduced form by glutathione reductase (GR) protecting the cell against excessive generation of damaging ROS (7). It is involved in oocyte activation and maturation and development of preimplantation embryos during ART (8).

The developing oocyte is surrounded by complex microenvironment consisting of granulosa, theca cells, and follicular fluid (FF) to form its microenvironment. FF is formed from plasma proteins and through the secretory function of the granulosa and theca cells (9, 10). It is suspected that biochemical specifications of the FF and its ROS threshold affect the oocyte quality, fertilization, embryo development, and pregnancy rate (11, 12). Moreover, the compositions of FF and blood are reciprocally affected by each other (13, 14). It is also suggested that FF is naturally supplied by powerful antioxidant systems to protect the oocyte from OS. Therefore, elevated levels of antioxidants in FF or serum are accompanied by increased chances of high-quality oocytes-retrieved ART and high-quality embryo production (14, 15). Vignini and colleagues showed that the free radical nitric oxide concentrations in FF is significantly higher in in vitro fertilization (IVF)-produced embryos with severe fragmentations, which shows its detrimental impact on embryo quality (16). In a recent study, the negative effect of total non-enzymatic antioxidant capacity and 8-hydroxy-2'-deoxyguanosine in the FF on the number of good-quality embryos was reported (17).

To the best of our knowledge, all relevant studies carried out so far have collected and examined the entire bulk of FF drawn upon ovum pick-up. In the present study, however, only the FF of the first aspirated follicle was examined in order to achieve more precise observations and results on the oocyte maturation stages and their correlations with enzyme levels and activities. Our aim was to show the correlation between the maturation stages of oocytes/embryos and GSH detoxification capacity in FF and serum separately. Moreover, due to difficult FF accessibility, we investigated whether the GSH levels in the serum represented those in the FF and therefore if serum could be used as an indicator of antioxidant activities in the FF.

## 2. Materials and Methods

### Materials

GR, tert-butyl hydroperoxide (t-BuOOH), and bovine serum albumin (BSA) were purchased from Sigma Chemical Co. (Poole, Dorset, UK); Na2-NADPH, di-sodium hydrogen phosphate (anhydrous) were obtained from Fluka Chemical Co. (Buchs, Switzerland). Sodium azide, sodium chloride, magnesium chloride, and EDTA were obtained from Merck (Darmstadt, Germany). Potassium chloride, potassium dihydrogen orthophosphate were from Fluka-England.

### Subject selection

This cross-sectional study was conducted at the infertility center of Ghadir-Mother and Child Hospital affiliated to the Shiraz University of Medical Sciences, Shiraz, Iran. All infertile PCOS women candidates for ART during February 2013- December 2014 were considered to be enrolled in this study. PCOS was diagnosed according to the revised 2003 Rotterdam criteria (1). Women aged more than 40 yr, diabetics, smokers, or those who refused to participate were excluded from the study. The couples with simultaneous male factor infertility were also excluded according to the WHO criteria (18).

### Ovarian stimulation protocol

Ovarian stimulation was performed using the long gonadotropin-releasing hormone (GnRH) agonist protocol. Downregulation was done by subcutaneous injection of GnRH agonist (0.3 mg buserelin, suprefact, serono, Switzerland) starting from the 21st day of the menstrual cycle. One ampule of highly purified urinary FSH (Fostimon,75 IU, SC, IBSA pharmaceutical, Italy) and one ampule of human menopausal gonadotropin (HMG) (Merional,75 IU, IM, IBSA pharmaceutical, Italy) were started from the second day of the cycle. The ovarian response to gonadotropins was monitored and adjusted by transvaginal ultrasonography (TVU), and plasma estradiol (E2) levels were checked every three days starting from the sixth day of the cycle. Gonadotropins injections were continued until the time at least two follicles reached 17-18 mm in diameter, while human chorionic gonadotropin (HCG, 10000 IU, Pregnyl, Merk, Germany) was injected for inducing the final oocyte maturation phase. The follicles were picked up 34-36 hr after the HCG injection under TVU guide. Just prior to the oocyte retrieval, 5 ml blood was taken and after centrifugation, serum was frozen at -20°C. The FF of the first aspirated follicle was collected separately in a sterile tube without any additional culture medium. The other follicles were collected in standard culture medium for conducting the routine IVF procedure.

The first-retrieved FF samples that contained more than one oocyte, had no oocyte, or those that became bloody were excluded from the study. This process was carried on until 80 acceptable FF samples were collected. The first aspirated oocytes were graded as GV, MI, or MII according to the standard classifications (19) were placed in standard culture media and monitored for their ongoing development after conventional IVF. Conventional IVF represents standard insemination, which involves combining sperm and egg outside the body in the laboratory and transferring the resulting embryo(s) back into the uterus. Three days post-oocyte retrieval, the resulting embryos were assessed as I, II, and III, where I indicated the highest and III showed the lowest embryo quality (20).

### Collection and preparation of FF

The samples in separated sterile containers were centrifuged at 200 × g for 5 min to remove cellular component. The clear supernatants were divided into aliquots and frozen at -20°C. The samples were used for measurement of OS parameters and total protein concentration.

### Determination of total protein concentration 

Biochemical evaluations were performed at the Biochemistry Department of Shiraz University of Medical Sciences. Total protein concentrations were determined to compare the levels of GSH, GPx, and GR among the different oocyte/embryo-quality groups to eliminate any bias related to the number of cells in each group. Total protein concentrations were measured using the Bicinchoninic acid assay (BCA) (Pierce, Rockford, IL). Bovine serum albumin was used as a standard protocol.

### Determination of GPx and GR activity 

GPx enzyme reduces and detoxifies the peroxides by GSH in-vivo. During this reaction, GSSG is generated. GSSG is recycled to reduced form by GR enzyme coupled with a reaction that converts NADPH to NADP${}^{+}$ which is associated with a decrease in absorbance at 340 nm. The amount of decreased absorption at this wavelength can be used to measure the activity of GPx, and the amount of consumed NADPH can be considered as an indicator of GR enzyme activity. GPx activity was assayed according to the Fecondo and Augusteyn procedure with some minor modifications (21). The procedure monitors the continuous regeneration of GSH from GSSG on a UV/vis spectrophotometer at 340 nm. T-BuOOH was used as substrate in the presence of GSH (0.1 mM), NADPH (0.15 mM), EDTA (3 mM), GR (2 U/ml), and phosphate buffer (pH = 7.2, 50 mM). Sample volume was 50 µl in total volume of 1 ml. The absorbance of the reaction products was observed after 5 min at 340 nm.

“GR activity was assayed by a previously described method (21). It was performed in a cuvette in a total volume of 1 ml that included 60 μM buffer, 5 mM EDTA (pH 8.0), 0.033 M GSSG, 2 mM NADPH, and the sample. The decrease in absorbance, which reflects the oxidation of NADPH during reduction of GSSG by the sample's GR, was monitored spectrophotometrically at 340 nm for 3 min. Results were based on a molar extinction coefficient for NADPH of 6.22 × 10${}^{6}$M${}^{-1}$cm${}^{-1}$. One unit of GR is defined as mU/mg cell protein".

### Determination of the total intracellular reduced GSH

GSH assay was performed by standard method (22). The standard curve was generated using a 1 mM solution of GSH and analyzed for GSH levels. Also, 2.3 ml of potassium phosphate buffer (0.2 M, pH 7.6) was added to 0.2 ml of the FF or serum samples, followed by adding 0.5 ml of DTNB (0.001 M) in a buffer. The absorbance of the reaction products was observed after 5 min at 412 nm. Total protein concentration for each sample was determined; GSH levels were expressed as nmol/mg protein.

### Ethical consideration

Before enrolment an informed consent form was signed by all of the couples participating in this study. The study was approved by the Institutional Research Ethics Board (Code: EC-P-92-4622).

### Statistical analysis

Kolmogorov-Smirnov test was used to evaluate whether the sample data was normally distributed. Paired-*t* test was used for comparing the means of each variable between serum and FF. In order to compare the means of variables in the serum and FF according to the different oocyte and embryo grades, Kruskal-Wallis test followed by pairwise multiple comparison of the data was performed by SPSS 16.0 (Statistical Package for the Social Sciences, IBM, Armonk, USA). P < 0.05 was considered statistically significant.

## 3. Results 

**Figure 1:**
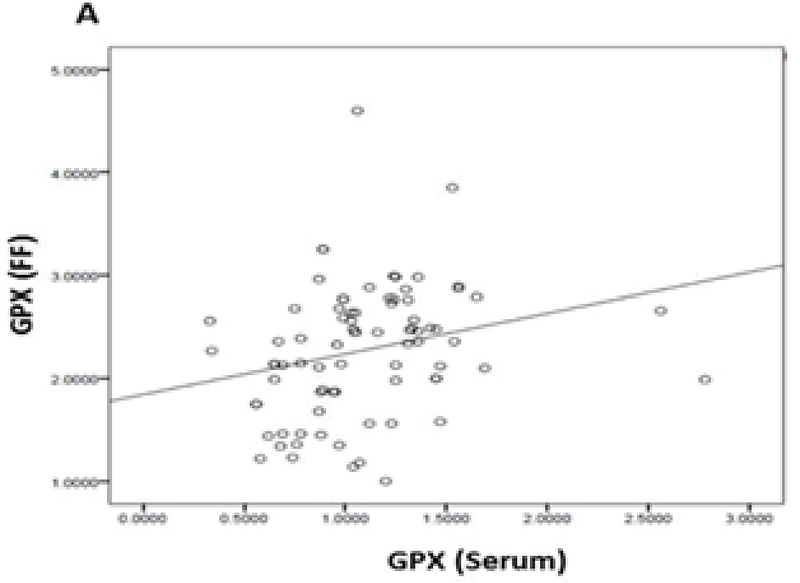
Correlations between the mean levels of GPx activity in the serum and FF of the first-retrieved oocyte.

**Figure 2:**
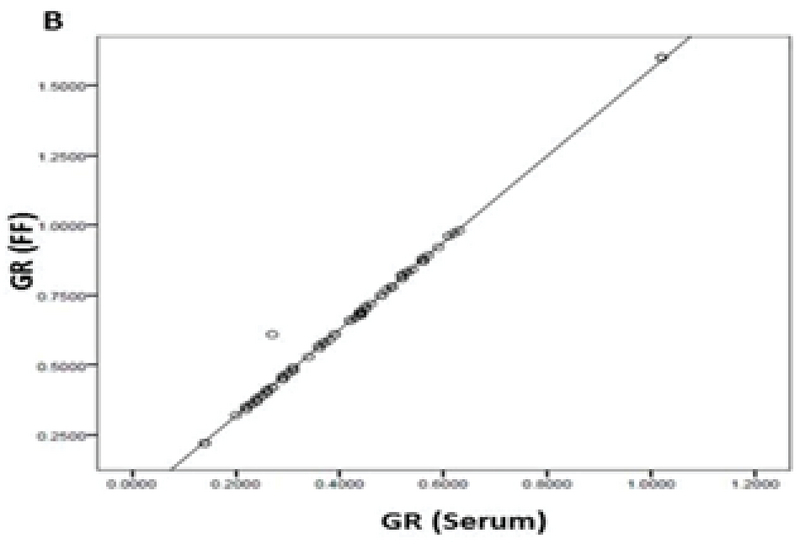
Correlations between the mean levels of GR activity in the serum and FF of the first-retrieved oocyte.

**Figure 3:**
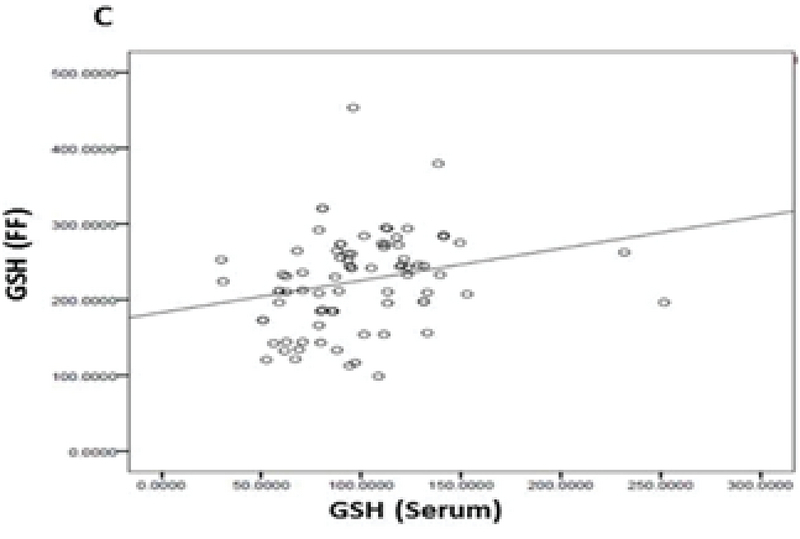
Correlations between the mean levels of GSH levels in the serum and FF of the first-retrieved oocyte.

Eighty PCOS women with a mean age of 29.83 ± 5.83 yr who were candidated for ART were enrolled in this study. Following ovum pick-up, the first oocytes retrieved were graded and followed for development; 53 out of 80 oocytes (66.25%) were graded as MII, 17 (21.25%) were MI, and 10 (12.5%) were GVs. Conventional IVF led to the production of 42 (52.50%) grade I embryos and 11 (13.75%) grade II embryos; 27 oocytes (33.75 %) failed to be fertilized. No grade III embryos were produced. Pearson correlation coefficient showed a significant positive correlation of the mean values of GPx (U/gr pro) (r = 0.249, p < 0.026) and GR (U/gr pro) (r = 0.996, p < 0.001) activities and GSH levels (nmol/mg pro) (r = 0.246, p = 0.028) in the FF and serum(Figures 1, 2, and 3). The mean values for GPx and GR activities and GSH levels in the FF were compared with these values in their corresponding serum (Table I).

The mean GPx and GR activity and GSH levels were significantly higher in the FF of the MII oocytes. The mean GPX activity and GSH levels in the serum samples did not show significant statistical difference among different oocyte grades but the mean serum GR activity was significantly higher for MII oocytes compared to GVs (Table II).

GPX activity and GSH levels were significantly higher in the serum and FF of the high-quality embryos with grade I compared to grade II embryo or no embryo group (Table III). Moreover, the mean of GR activity was significantly higher in the serum and FF of grades I and II embryos compared to no embryo group. Examination of serum and FF of grades I and II embryos revealed no significant difference in their GR activities (Table III).

**Table 1:** Comparison of the mean values for GPx, GR activities, and GSH levels between the serum and FF of the first-retrieved oocyte


	**GPX (U/gr pro)**	**GR (U/gr pro)**	**GSH (nmol/mg pro)**
**FF**	2.27 ± 0.63	0.64 ± 0.24	224.31 ± 62.34
**Serum**	1.09 ± 0.39	0.41 ± 0.15	98.39 ± 0.36.33
**P-value**	< 0.001*	< 0.001*	< 0.001*
Values are represented as Mean ± SD; *P-value < 0.05 is statistically significant FF: Follicular fluid; GPx: Glutathione peroxidase; GR: Glutathione reductase; GSH: Reduced form of glutathione The mean values are compared by paired *t* test			

**Table 2:** GPx and GR activities and GSH levels in the serum and FF of the first-retrieved oocytes at different maturation stages


	**Sample**	**GV (n = 10)**	**MI (n = 17)**	**MII (n = 53)**	**P-value**
	FF	2.00 ± 0.89	1.99 ± 0.52	2.41 ± 0.56${}^{▴,▪}$	0.012
**GPx (U/gr pro)**	Serum	1.08 ± 0.59	1.06 ± 0.38	1.1 ± 0.36	0.563
		FF	0.49 ± 0.09	0.55 ± 0.19	0.7 ± 0.25${}^{▴}$	0.006
**GR (U/gr pro)**	Serum	0.31 ± 0.05	0.35 ± 0.12	0.44 ± 0.16${}^{▴}$	0.008
		FF	197.47 ± 88.14	196.71 ± 51.96	238.23 ± 56.07${}^{▴,▪}$	0.012
**GSH (nmol/mg pro)**	Serum	97.80 ± 54.21	93.33 ± 35.47	100.12 ± 33.05	0.531
	Data presented as Mean ± SD; P-values ≤ 0.05 were considered significant; ${}^{▴}$Statistically significant between GV and MII; ${}^{▪}$Statistically significant between MI and MII FF: Follicular fluid; GPx: Glutathione peroxidase; GR: Glutathione reductase; GSH: Reduced form of glutathione The mean values are compared by paired *t* test					

**Table 3:** GPx and GR activities and GSH levels in the serum and FF of the resulting embryos of different grades


	**Sample**	**No embryo (n = 27)**	**Grade II (n = 11)**	**Grade I (n = 42)**	**P-value**
	FF	1.99 ± 0.66	2.00 ± 0.50	2.52 ± 0.53${}^{▴,▪}$	0.001
**GPx (U/gr pro)**	Serum	1.07 ± 0.46	0.97 ± 0.61	1.14 ± 0.26${}^{▴,▪}$	0.016
		FF	0.53 ± 0.16	0.72 ± 0.45${}^{*}$	0.69 ± 0.18${}^{▴}$	0.005
**GR (U/gr pro)**	Serum	0.33 ± 0.10	0.46 ± 0.29${}^{*}$	0.44 ± 0.11${}^{▴}$	0.004
		FF	196.97 ± 65.96	197.47 ± 50.14	248.91 ± 23.02${}^{▴,▪}$	0.001
**GSH (nmol/mg pro)**	Serum	94.98 ± 42.52	88.25 ± 55.89	103.23 ± 24.00${}^{▴,▪}$	0.012
	Data presented as Mean ± SD; P-values ≤ 0.05 were considered significant; ${}^{▴}$Statistically significant between no embryo and grade I; ${}^{▪}$Statistically significant between grade II and grade I; ${}^{*}$Statistically significant between no embryo and grade II FF: Follicular fluid; GPx: Glutathione peroxidase; GR: Glutathione reductase; GSH: Reduced form of glutathione The mean values are compared by paired *t* test					

## 4. Discussion

The microenvironment of the oocyte determines its developmental potential. In this study, we measured GPx and GR activities and GSH levels in the serum and FF of the first-retrieved oocytes of PCOS women who underwent IVF. Subsequently, the oocytes and resulting embryos were graded according to their quality.

GPx, GR, and GSH are antioxidants that scavenge the free radicals and lipid peroxides to maintain the intracellular balance (23). Our results indicated that the mean levels of all of the antioxidants were higher in the FF compared to serum with a positive correlation between these two biological samples. Similarly, Leroy and colleagues demonstrated that changes of all metabolites in the serum of dairy cows were accompanied by similar changes in their FF especially for glucose and urea (13). However, to the best of our knowledge, this is the first time that the correlation between the serum changes of the GSH-related antioxidants and first-retrieved FF is evaluated.

The biochemical composition of FF has a direct effect on the maturation ability of oocyte and evidence shows that the GSH antioxidant system in FF plays an important role in oocyte maturation and subsequent IVF outcome (12, 24). For instance, reduced OS when the ovarian stimulation was associated with micronutrients supplementation improved the number of good-quality oocytes (25). Kish and colleagues showed that the transcript levels of thioredoxin (a regulating protein of reduction-oxidation reactions) in FF of oocyte-containing follicles was higher compared to empty follicles (26). In agreement with these studies, our study showed that higher GPx and GR activities and GSH levels in the FF were associated with higher oocyte quality, and there was an increasing trend correlating with better oocyte quality.

However, despite an increasing trend in the presence of more developed first-retrieved oocytes, no significant difference was observed in their corresponding serum GPx and GSH levels, except for GR activity which recorded statistically significant values in the serum of MII oocytes when compared to that of GVs.

We hypothesized that GSH-related metabolites in serum played as direct indicators of oocyte quality, which obviously is much more available compared with FF. Nevertheless, our data did not affirm the whole idea. It is worth mentioning that oocyte is in direct contact with its own FF metabolites and therefore oocyte quality and their FF antioxidant levels are tightly related, while the serum antioxidant levels reflect the metabolic condition of the whole body. Moreover, in addition to serum and FF antioxidants capacity, oocyte and embryo are protected by other antioxidant systems (27).

The adequate antioxidant concentrations influence the fertilization rate and are essential for proper embryo development (12, 28, 29). There is a cut-off value for ROS in the FF. Values greater than the cut-off are considered toxic for the embryo formation and negatively impact the pregnancy outcomes (14, 15). It has been shown that in patients with low fertilization rates and low-quality blastocysts, total GSH levels were lower (12). In another study, Vignini and colleagues showed that the concentration of free radical nitric oxide in FF was significantly higher in IVF-produced embryos with severe fragmentations, confirming its detrimental role in embryo quality (16). In the present study, mean levels for serum and FF GPx activity and GSH were significantly higher in grade I embryos compared to grade II and no-embryo groups. The mean GR activity in serum and FF of patients undergone IVF cycle with resulting embryos were significantly higher than no embryo group. These data affirm the role of GSH antioxidant system in the production of good-quality embryos.

To the best of our knowledge, this study is the first to examine the FF of the first-retrieved follicle to demonstrate the association between elevated follicular GPx and GR activities and GSH levels with higher quality oocytes and embryos. In addition, higher serum antioxidant levels were associated with higher embryo qualities in IVF cycles.

## 5. Conclusion

According to our results, GSH -dependent antioxidant system acts more efficiently in the advanced maturation stages of oocytes and embryos and the antioxidants quantities in the FF can be potential predictors of oocyte and embryo quality.

##  Conflict of Interest

The authors declare that they have no conflict of interest.
